# Expression of the VP2 Protein of Murine Norovirus by a Translation Termination-Reinitiation Strategy

**DOI:** 10.1371/journal.pone.0008390

**Published:** 2009-12-22

**Authors:** Sawsan Napthine, Robert A. Lever, Michael L. Powell, Richard J. Jackson, T. David K. Brown, Ian Brierley

**Affiliations:** 1 Division of Virology, Department of Pathology, University of Cambridge, Cambridge, United Kingdom; 2 Department of Biochemistry, University of Cambridge, Old Addenbrookes Site, Cambridge, United Kingdom; Victor Chang Cardiac Research Institute (VCCRI), Australia

## Abstract

**Background:**

Expression of the minor virion structural protein VP2 of the calicivirus murine norovirus (MNV) is believed to occur by the unusual mechanism of termination codon-dependent reinitiation of translation. In this process, following translation of an upstream open reading frame (ORF) and termination at the stop codon, a proportion of 40S subunits remain associated with the mRNA and reinitiate at the AUG of a downstream ORF, which is typically in close proximity. Consistent with this, the VP2 start codon (AUG) of MNV overlaps the stop codon of the upstream VP1 ORF (UAA) in the pentanucleotide **UAA** UG.

**Principal Findings:**

Here, we confirm that MNV VP2 expression is regulated by termination-reinitiation and define the mRNA sequence requirements. Efficient reintiation is dependent upon 43 nt of RNA immediately upstream of the **UAA** UG site. Chemical and enzymatic probing revealed that the RNA in this region is not highly structured and includes an essential stretch of bases complementary to 18S rRNA helix 26 (Motif 1). The relative position of Motif 1 with respect to the **UAA** UG site impacts upon the efficiency of the process. Termination-reinitiation in MNV was also found to be relatively insensitive to the initiation inhibitor edeine.

**Conclusions:**

The termination-reinitiation signal of MNV most closely resembles that of influenza BM2. Similar to other viruses that use this strategy, base-pairing between mRNA and rRNA is likely to play a role in tethering the 40S subunit to the mRNA following termination at the VP1 stop codon. Our data also indicate that accurate recognition of the VP2 ORF AUG is not a pre-requisite for efficient reinitiation of translation in this system.

## Introduction

For most eukaryotic mRNAs, translation initiation is a 5′-end-dependent process beginning with recognition of the cap structure by the cap-binding complex eIF4F [1] and (usually) recognition of the AUG codon of the first open reading frame (ORF) on the mRNA by the scanning ribosome complex [2]. This 5′-end dependence is a problem faced by many RNA viruses with polycistronic genomes and elaborate strategies have been developed to facilitate access of ribosomes to downstream open reading frames (ORFs). Amongst these, a number of unconventional translation strategies have been described [3]. These include leaky scanning of 40S subunits past the start codon of the first ORF [4], the possession of intercistronic internal ribosome entry signal [5], programmed ribosomal frameshifting during elongation [6] and stop codon suppression at the termination step [7]–[8]. Another strategy that has evolved to allow expression of a downstream ORF is termination-reinitiation (also referred to here as stop-start). In this process, ribosomes translate the upstream ORF but following termination, a proportion of 40S subunits remain tethered to the mRNA and go on to reinitiate at the start codon of the downstream ORF. This termination-dependent reinitiation strategy allows the coupled expression of products from adjacent ORFs and thus the production of a defined ratio of gene products.

Termination-reinitiation in virus systems [9] was first described in the synthesis of the BM2 protein of the orthomyxovirus influenza B virus [10] and subsequently in expression of VP2 of feline calicivirus (FCV) of the genus *Vesivirus* [11]–[13] and VP10 of the calicivirus rabbit haemorrhagic disease virus (RHDV) of the genus *Lagovirus* [14]. A related phenomenon is also seen in expression of the M2-2 protein [15]–[16] of the paramyxovirus respiratory syncytial virus (RSV) and the M2-2 protein [17] of pneumovirus of mice (PVM). In FCV, the stop codon (UGA) of the major capsid stop-start protein VP1 overlaps the start codon of the minor capsid protein VP2 ( A**UG** **A** ) (the stop-start “window”). Efficient termination-reinitiation depends upon several factors, including the close proximity of the stop and start codons, the transit of ribosomes along the VP1 mRNA up to the stop codon and a stretch of some 70–80 nucleotides (nt) of mRNA upstream of the stop-start window whose primary sequence, rather than the encoded protein, is key. This region of the mRNA, termed the termination upstream ribosomal binding site (TURBS), is needed for the retention of post-termination 40S subunits [11]. A short sequence of the TURBS (termed Motif 1) that is complementary to part of helix 26 of 18S rRNA likely acts to tether the 40S ribosomal subunit to the mRNA post-termination, allowing time for the ribosome to acquire the factors necessary to initiate on the downstream ORF [12], [14], [18]. The TURBS may also act by recruitment of eukaryotic initiation factor 3 (eIF3) or eIF3/40S complexes [13]. Recent studies of termination-reinitiation in the expression of the orthomyxovirus influenza BM2 protein have revealed a requirement for a shorter stretch of mRNA (45 nt) upstream of the stop-start window, but nevertheless, the RNA contains a similar TURBS Motif 1 [19]. From RNA secondary structure probing, it has been proposed that this stretch may be displayed on the apical loop of a stem-loop structure that may form following transit of the ribosome through the region and termination at the upstream ORF stop codon [9], [19].

In this paper, we describe an analysis of termination-reinitiation in the expression of the VP2 protein of murine norovirus (MNV), a calicivirus of the genus *Norovirus*. The VP2 start codon (AUG) of MNV overlaps the stop codon of the upstream VP1 ORF (UAA) in the pentanucleotide **UAA** UG , consistent with a termination-reinitiation strategy, and a stretch of bases (5′ UAUGGGAA 3′) complementary to 18S rRNA helix 26 is present upstream. Using a luciferase-based reporter plasmid, we show that VP2 is expressed by termination-reinitiation and provide evidence consistent with a functional interaction between the coding region of the VP1 mRNA and 18S rRNA. The formation of mRNA secondary structure within the TURBS is also investigated. Overall, our data suggest that the mechanism of VP2 expression is broadly similar to that of the other caliciviruses and influenza B. However, in contrast to what was observed with the FCV signal [13] and seen here with influenza BM2, termination-reinitiation at the MNV signal shows resistance to the initation inhibitor edeine. Thus the mechanism by which the AUG of the downstream ORF is recognised may differ.

## Results

### The Murine Norovirus VP2 Protein Is Translated via Termination-Dependent Reinitiation

**Figure 1:**
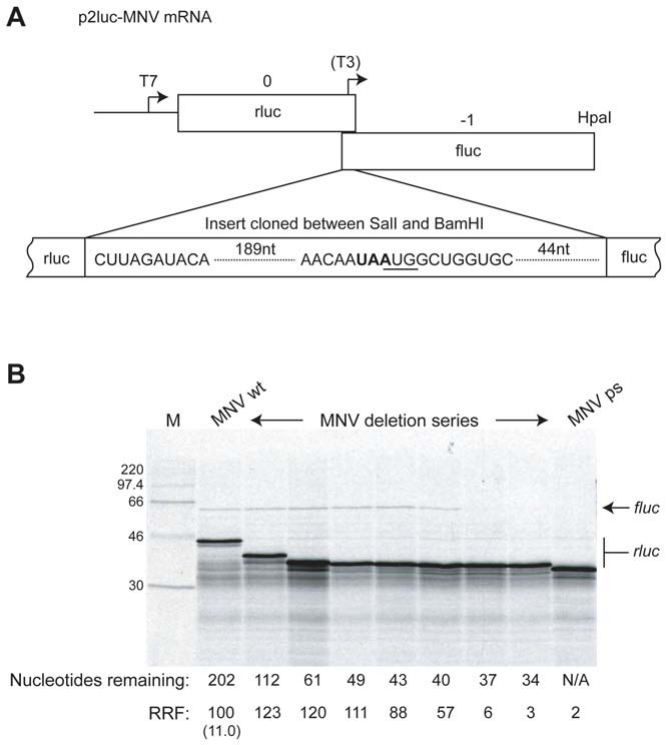
Minimal sequence requirements for MNV termination-reinitiation. A) Schematic of the p2luc-MNV reporter mRNA. The termination-reinitiation region (203 nt upstream and 52 nt downstream of the **UAA** UG motif) was cloned into the *SalI* and *BamHI* sites of the p2luc reporter plasmid. *HpaI* run-off transcripts for *in vitro* translation were generated using T7 RNA polymerase. The location of the T3 promoter present in the structure mapping construct p2luc-MNV-T3 is indicated. B) Deletion analysis of MNV termination-reinitiation. A series of p2luc-MNV variants were prepared with stepwise, in-frame deletions from the 5′ end of the inserted viral sequence. The wild-type (wt), premature stop (ps) and deletion mutant plasmids were linearised with *HpaI* and run-off transcripts translated in Flexi® RRL at a final RNA concentration of 50 µg/ml in the presence of [^35^S]-methionine and 140 mM added KCl. The products were resolved by 12% SDS-PAGE and visualised by autoradiography. The number of nucleotides of viral sequence remaining up to the AUG start codon of the MNV ORF is shown below the gel. The product of the full-length or truncated versions of the rlucVP1 ORF (predicted size of MNVwt is 42 kDa) is marked rluc, and the VP2fluc product (predicted size, 62 kDa) is marked fluc. The MNV ps rluc product is the shortest (predicted size, 33 kDa). RRF denotes the relative reinitiation frequency in comparison to MNVwt (set at 100). The figure in brackets represents the ratio of the intensity of the fluc and rluc products (adjusted for methionine content and expressed as a percentage) for the MNVwt mRNA.

To investigate termination-reinitiation in the synthesis of the MNV VP2 protein, a 255 bp fragment of viral cDNA was cloned between the *SalI* and *BamHI* sites of the dual-luciferase reporter vector p2luc [20]. The cloned fragment, which contained 203 bp of sequence information upstream of the **UAA** UG stop-start window, and 52 bp downstream was suspected, on the basis of work with other viruses (see Introduction), to contain all of the required sequences for termination-reinitiation. The cDNA fragment was cloned in such a way that the *Renilla* and Firefly luciferase ORFs were in frame with the stop and start codons respectively of the termination-reinitiation motif to give an ORF configuration 5′ rlucVP1-VP2fluc 3′ (Figure 1). This vector, named p2luc-MNVwt, contains a T7 RNA polymerase promoter allowing synthetic mRNAs to be generated to investigate the stop-start process in *in vitro* translation reactions.

The translation of *in vitro* synthesised wild-type (wt) mRNA from p2luc-MNVwt was carried out in Flexi® rabbit reticulocyte lysate (Flexi®RRL) supplemented with 140 mM KCl (see Materials and Methods) and gave products of the expected sizes (upstream rlucVP1 ORF, ∼42 kDa, downstream VP2fluc ORF, ∼64 kDa, Figure 1b). The molar ratio of VP2fluc to rlucVP1 (taking into account the methionine content of the two proteins) was typically in the region of 1∶10. Thus, initiation on the downstream ORF occurred at a frequency of about 10% of that of the upstream ORF. That this was indeed the product of the second ORF was further confirmed by comparing the migration of RRL translation products from mRNAs derived from p2luc-MNVwt that had been linearised at different points within the second ORF (data not shown). Termination-reinitiation is distinct from IRES-mediated expression of downstream ORFs as translation through the upstream ORF is an absolute requirement [11], [14], [16]. In order to establish whether this is also the case for MNV expression, a premature in-frame stop-codon was inserted close to the end of the rluc ORF but upstream of VP1 sequence information (219 bp upstream of the authentic rlucVP1 termination codon). If the expression of VP2fluc is a result of termination-reinitiation, translating ribosomes would be unable to reach the AUG start codon of VP2fluc in the mutant mRNA and the ORF could not be translated. As is clear in Figure 1b, the introduction of a premature stop codon into the rluc/M1 ORF abolished expression of the VP2/fluc product, but had no effect on synthesis of the upstream ORF (rlucVP1ps, ∼33 kDa). These data are thus consistent with a termination-reinitiation strategy for the expression of the VP2 protein and confirm a requirement for translation through the upstream ORF.

### Expression of MNV VP2 Is Dependent on ∼40–43 nt Upstream of the UAAUG Motif

Previous work has suggested that viral termination-reinitiation events show little dependence on sequence information downstream of the “stop-start” window but require 45–250 nt of upstream primary sequence [11,14,16.19]. In order to determine the minimal sequence requirements for termination-reinitiation in VP2 expresssion, deletions of increasing size were made from the 5′ end of the inserted viral information (Figure 1b). The stop-start product was synthesised efficiently with up to 43 nt of VP1 information present upstream of the **UAA** UG motif, and to a lesser extent with 40 nt. However, deletion to 37 nt or less abolished expression of the termination-reinitiation product (Figure 1b). These data indicate that only 40 nucleotides of VP1 primary sequence immediately upstream of the stop-start window are required for termination-reinitiation *in vitro*, although 43 nt are required for full activity.

### Termination-Reinitiation of MNV VP2 Synthesis Is Dependent upon an mRNA Sequence with Complementarity to 18S rRNA

**Figure 2:**
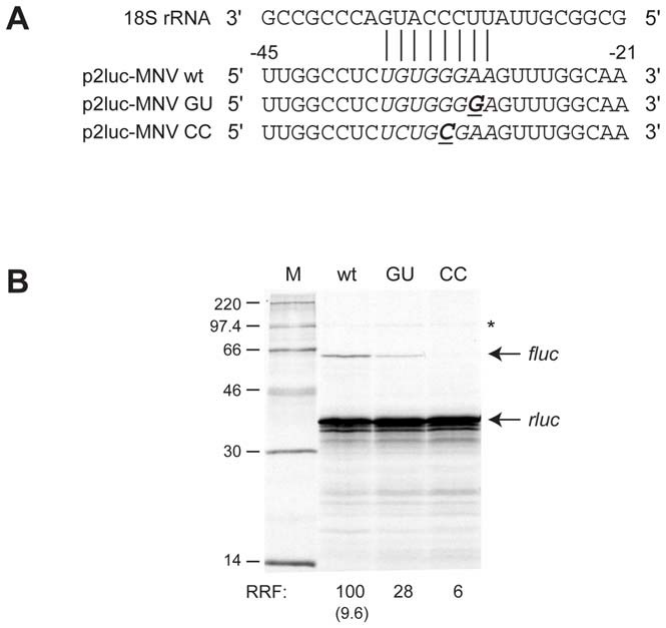
Investigating the role of the MNV 18S rRNA complementary region (Motif 1) in termination-reinitiation. A) Comparison of part of the sequence of helix 26 of 18S rRNA and the complementary sequence present upstream of the termination-reinitiation site of MNV. Contiguous nucleotides complementary to the 18S rRNA are shown in *italics*. Putative mRNA-rRNA base pairing is marked, with the mRNA bases numbered relative to the stop codon of rlucVP1. The sequence of the two constructs generated to address the role of the complementary region is also shown, with changes in bold and underlined. B) Plasmids were linearised, transcribed, translated and analysed according to the legend of Figure 1. Lanes are labelled with the last two letters of each reporter plasmid name.

In FCV, RHDV and influenza B, it has been shown that termination-reinitiation requires a closely conserved primary sequence element (referred to as Motif 1) that is complementary to a region of helix 26 of 18S rRNA [11]–[12], [14]). The position of Motif 1 varies somewhat, with the 5′ base 73 nt (RHDV), 63 nt (FCV) or 34 nt (influenza B) upstream of the stop codon of the first ORF. Mutational analysis has revealed that this sequence is essential for the stop-start process [12]–[14], [18]–[19]. Within the ∼43 nt minimal region of the MNV VP1 RNA required for VP2 expression, a stretch of bases with a similar level of complementarity to 18S rRNA is also found (Figure 2a, complementary bases are shown in italics). To investigate whether this region plays a role in termination-reinitiation in VP2 expression, two point mutations were made to disrupt potential mRNA:rRNA pairs (Figure 2a). In the first, the A at –31 was mutated to a G (p2luc-MNV GU), creating a presumably slightly weaker putative U-G base pair between the rRNA and mRNA. In the second, the G at –32 was changed to a C (p2luc-MNV CC), which would act to disrupt the interaction between 18S rRNA and mRNA. As can be seen in Figure 2b, the latter mutation greatly reduced expression of the VP2fluc product, supporting the idea that an interaction between the 18S rRNA and the mRNA just upstream of the termination-reinitiation site is required. In the mutant where pairing was predicted to be maintained (p2luc-MNV GU) termination-reinitiation was clearly detectable, although the efficiency was reduced somewhat compared to that of wild-type mRNA.

### RNA Secondary Structure Analysis of the Region Required for Termination-Reinitiation in MNV VP2 Synthesis

**Figure 3:**
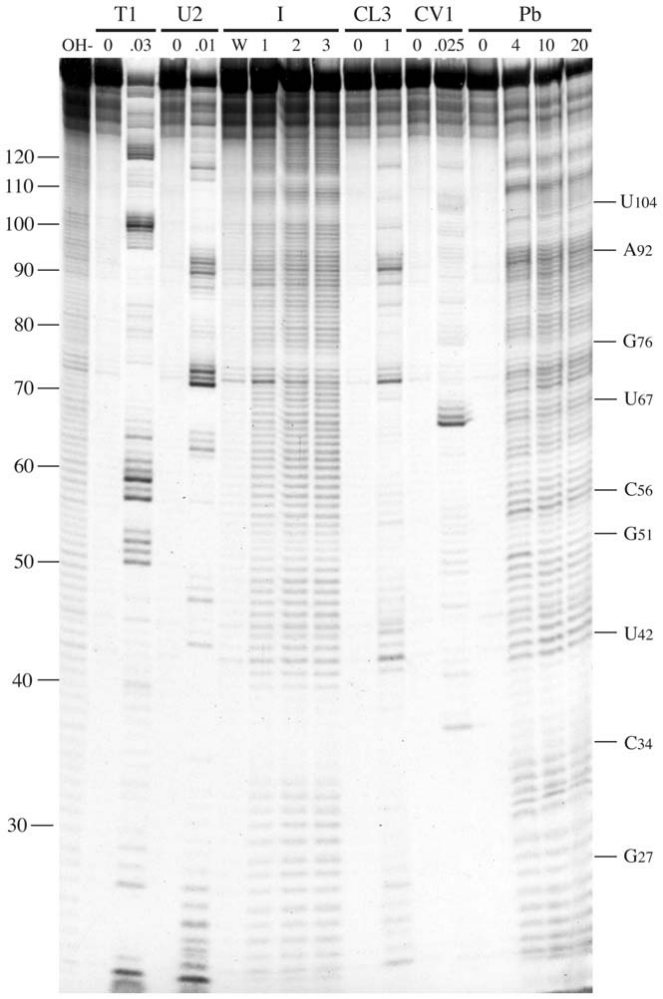
Structure probing of the MNV termination-reinitiation signal. RNA derived by transcription of p2luc-MNV-T3/*BamHI* with T3 RNA polymerase was 5′ end-labelled with [γ-^33^P]-ATP and subjected to limited RNase or chemical cleavage using structure-specific probes. Sites of cleavage were identified by comparison with a ladder of bands created by limited alkaline hydrolysis of the RNA (OH-) and the position of known RNase U2 and T1 cuts, determined empirically. Products were analysed on a 10% acrylamide/7M urea gel containing formamide. Data was also collected from 6% and 15% gels (gels not shown). Enzymatic structure probing was with RNases T1, U2, CL3 and CV1. Uniquely cleaved nucleotides were identified by their absence in untreated control lanes (0). The number of units of enzyme added to each reaction is indicated. Chemical structure probing was with imidazole (I, hours) or lead acetate (Pb; mM concentration in reaction). The water lane (W) represents RNA which was dissolved in water, incubated for four hours and processed in parallel to the imidazole-treated sample. The sequence of the probed RNA and the inferred secondary structure is shown in Figure 4.

**Figure 4:**
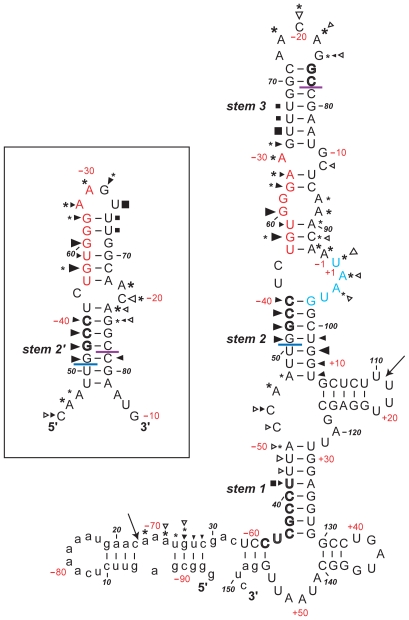
Summary of the MNV structure probing results. The sensitivity of bases in the MNV termination-reinitiation region to the various probes is shown for an *mfold* prediction (see text). The first base of the transcript is numbered 1. The bases are also numbered (in red) with respect to the VP1 stop codon (with the U of the UAA codon numbered +1, the preceding base numbered -1). The reactivies of the T1 (black triangle), U2 (asterisk), CL3 (open triangle) and CV1 (black square) probes are marked. The size of the symbols is approximately proportional to the intensity of cleavage at that site. Lead and imidazole cleavages are not marked, but bases resistant to cleavage by both reagents are shown in bold/outline font. The two large arrows show the boundaries beyond which no structure mapping information was obtained. The stretch of bases in red indicate the 18S rRNA complementary region. Bases that form the stop-start overlap are in blue. The blue line indicates the start of the minimal essential region required for efficient termination-reinitiation. The purple line indicates the likely location of the 5′-edge of a ribosome poised at the termination codon (UAA, in blue). Bases in lower case are of vector origin. The *mfold* shown in the box shows part of an alternative pairing possibility in which the 5′ arm of stem 2 pairs with a different region (to give stem 2′; see text).

The experiments described above confirm the existence of Motif 1 and its role in reintiation in MNV. It was therefore of interest to determine the context of this 18S rRNA complementary region within the global RNA secondary structure of the minimal functional sequence, and to compare the structure with that determined for the influenza BM2 signal [19]. To achieve this, a bacteriophage T3 promoter was inserted upstream of the viral sequence of the p2luc-MNV.61 plasmid (Figure 1b). The plasmid was linearised with *BamHI*, T3 run-off transcripts synthesised and the RNA end-labelled with [^33^P]-γATP. The labelled transcripts were subjected to limited chemical and enzymatic probing prior to analysis on denaturing polyacrylamide gels. The chemical probes used were imidazole and lead acetate, specific for cleavage of single stranded regions. Enzymatic probes were RNases T1, U2 and CL3, which preferentially cleave single-stranded G, A and C residues respectively, and RNase CV1, which cuts in helical regions in double-stranded or stacked conformations. A representative stucture mapping gel is shown in Figure 3 and in Figure 4, the data are mapped onto *mfold* predictions of the secondary structure of the “stop-start” region.

Structure probing analysis of the MNV signal revealed that, like the BM2 signal, the mRNA in the region essential for termination-reinitiation is not highly structured. This was especially evident from the chemical probes, with most residues sensitive to imidazole and lead cleavage. The enzymatic probes were also active against the majority of bases in the region and consistent with this, CV1 probing identified very few double-stranded or stacked bases. We also noticed a few CL3 cuts at residues other than C, although the reason for this is uncertain. Minimal free energy *mfold* predictions, performed using the online server of Zuker (http://mfold.bioinfo.rpi.edu/cgi-bin/rna-form1.cgi) indicated that the most stable RNA fold was the bulged stem-loop shown in Figure 4. However, the correspondence between this *mfold* and the mapping data was not absolute. Whilst in general, the single-stranded probes displayed more activity against regions of the model predicted to be single-stranded than they did against predicted helices, there were anomalies. For example, residues G51-52 were sensitive to RNase T1, yet were predicted to be in a double-stranded region (stem 2). Generally, the predicted duplexes showed more reactivity to single-stranded probes than one would expect for stable double-stranded stretches. Therefore, it seems likely that the RNA in this region is metastable, potentially adopting a number of co-existing structures. In our model, the sequence complementary to 18S rRNA is sequestered between two putative stems (stems 2 and 3; Figure 4) at a location similar to that found with BM2 [19]. Given that the termination-reinitiation process requires the ribosome to translate through the VP1 ORF, secondary structure in the RNA upstream of the “stop-start” window would be unwound and perhaps remodelled as the ribosome transits to the termination codon. Toeprinting of ribosomes paused at initiation codons has shown that the 5′ edge of the ribosome is some 12 to 13 nt from the first base of the AUG [21]. This would place the 5′ edge of the terminating ribosome (with the UAA codon in the A-site) close to residue C78 on our mRNA. Thus a terminating ribosome would prevent formation of the secondary structure, conceivably releasing the 18S rRNA complementary region for interaction with the ribosome (see Discussion). An alternative structure can be predicted under such circumstances, shown in the inset box in Figure 4. In this structure, the 5′ arm of the original stem 2 is predicted to pair with alternative bases to generate a new stem (stem 2′) with Motif 1 forming part of the apical loop. This alternative fold is attractive for a number of reasons. By displaying Motif 1 on an apical loop, this could promote 18S rRNA binding and ribosome tethering [19]. Until ribosomes transit through this region, Motif 1 would remain within a larger structure with potentially reduced access to the ribosome which could, at least in part, account for the observation that the signal does not appear to function as an IRES. The deletion analysis of Figure 1 is also consistent with a role for this alternative structure as the functional “end-point” maps to the start of the 5′ arm of stem 2′. Furthermore, most, if not all, viral TURBS have the potential for base-pairing between regions flanking Motif 1 [18]. Nevertheless, it should be noted that the structure mapping data are not fully consistent with this alternative structure, for example, there is considerable sensitivity to RNase T1 cleavage within the 5′ arm of stem 2′. This is considered further in the Discussion section.

### Effect of Moving the Stop Codon of rlucVP1 Further Downstream of the Start Codon of VP2fluc

**Figure 5:**
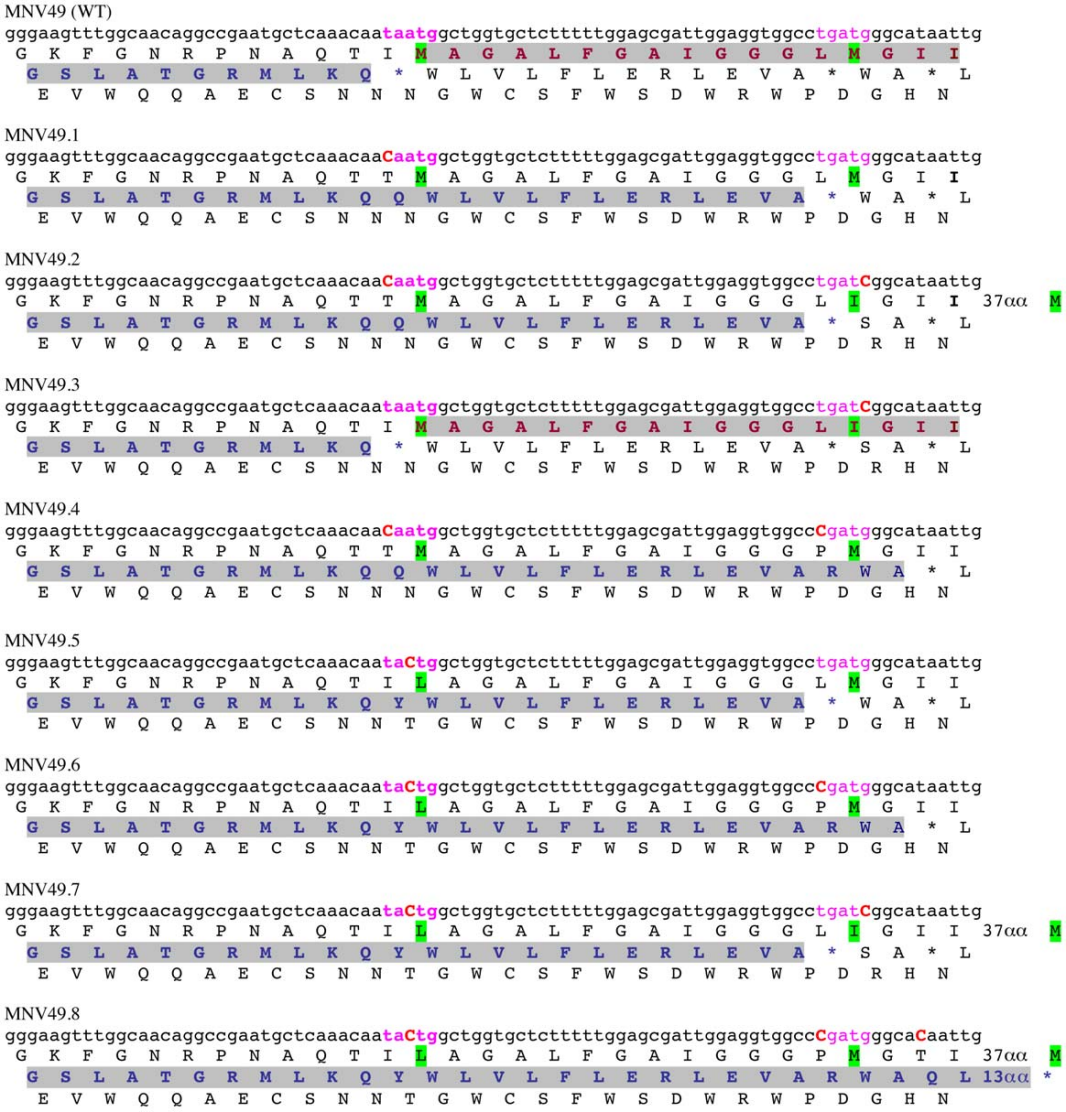
The effect of moving the stop codon of the termination-reinitiation window further downstream on the mRNA. A series of plasmid constructs were prepared, based on MNV.49 (a fully functional, truncated version of pMNVwt [see Figure 1] which acts as the “wild-type” reference construct [WT] in these experiments), in which the stop and start codons of the termination-reinitiation signal were altered. The figure shows the primary sequence and three-frame translation of the relevant region of the mRNA encoded by each construct. The natural stop-start motif is shown in pink and emboldened text, the downstream fortuitous stop-start motif in pink. Mutations within the mRNA sequence are highlighted by uppercase, red emboldened characters. The upstream rlucVP1 ORF is highlighted in grey, as is the downstream VP2fluc ORF where this is known. Likely key methionines (start codons) or their replacement amino acid are highlighted in green.

**Figure 6:**
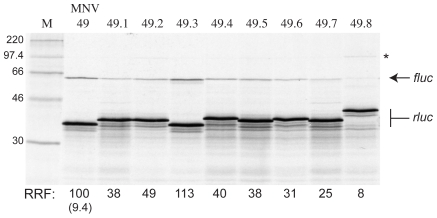
Effect of moving the stop codon of the termination-reinitiation window further downstream on the mRNA. The plasmid constructs of Figure 5 were linearised with *HpaI* and run-off transcripts translated and analysed as decribed in the legend to Figure 1. The product of the full-length or truncated versions of the rlucVP1 ORF is marked rluc, and the VP2fluc product (predicted size, 62 kDa) is marked fluc. The longer product observed in the 49.8 translation is asterisked.

Efficient termination-reinitiation seems to require the close proximity of the stop and start codons [12], [14], [19]. To investigate whether this is also the case for MNV, the authentic stop codon of rlucVP1 (in the context of the fully functional MNV49; see Figure 1b) was mutated from UAA to CAA such that the first ORF was extended by 13 amino acids (MNV49.1 Figure 5). The separation of stop and start codons by such a distance in BM2 is known to reduce reinitiation about 10-fold [19], but with the MNV signal, only a three-fold reduction in flucVP2 synthesis was observed (Figure 6). A possible explanation for this lies in the fact that the “new” stop codon is itself embedded within a second potential stop-start sequence (UGAUG) which could facilitate some reinitiation, but perhaps at a lower frequency, as it would not necessarily be spaced appropriately with respect to Motif 1. Another possibility is that 40S subunits terminating at the downstream stop-start sequence can reinitiate, at a reduced frequency, at the correct (upstream) AUG, despite the increased spacing, with the 40S subunit remaining tethered to the mRNA and “snapping-back” to the normal position of reinitiation. In an attempt to distinguish between these possibilities, additional constructs were prepared in which point mutations were introduced into pMNV.49 such that the termination and start codons in the two stop-start regions were changed separately and in combination (MNV49.2 to MNV49.8; see Figures 5 and 6). From this analysis, it is evident that modification of the authentic termination-reinitiation motif reduces reinitiation, irrespective of whether the stop or start codon is eliminated. Alteration of the AUG codon had the most effect, with reinitiation reduced to 8–38% of the wild-type level. When the natural termination site was changed such that termination now took place 13 or 15 amino acids downstream, the frequency of termination-reinitiation was also reduced, to 25–49% of the wild-type level. In MNV49.8, where termination of the upstream ORF occurred 30 amino acids downstream of the natural site, very little reinitiation was seen (8% of the wild-type level), indicating that the ribosome is unable to locate the authentic AUG from such a distal termination site. In the translation of this mRNA, an additional product was seen (asterisked in Figure 6) whose size is consistent with a fusion of the encoded ORFs (this is considered in the Discussion section). Reinitiation events that take place at either the authentic or the downstream stop-start motifs would produce polypeptides that differ in size by only 13 amino acids, thus we would not expect to be able to distinguish them by SDS-PAGE, and this is clear in Figure 6, where the reinitiation products show very similar electrophoretic mobilities. Thus we cannot say with confidence whether a particular AUG (or both) is used. However, the substantial reintiation activity displayed by MNV49.7, an mRNA in which both AUGs were changed, indicates that non-AUG codons can act as reinitiation codons, although probably at reduced efficiency. This is consistent with other work demonstrating that reinitiation can occur at non-AUG codons within the context of a termination-reinitiation signal [12], [14], [19]. Whilst in principle, reinitiation of translation of the MNV 49.7 VP2fluc ORF, following termination, could occur at the next available AUG, this is located 54 amino acids from the natural stop-start signal and initiation here would produce a substantially shorter product that would have been detectable by SDS-PAGE. Thus in this mRNA, a significant proportion of ribosomes (25% of the wild-type level) that terminate 13 amino acids downstream of the authentic stop-start site can reinitiate in an AUG-independent manner within the stop-start window.

### The MNV Termination-Reinitiation Signal Shows Resistance to the Action of the Initiation Inhibitor Edeine

**Figure 7:**
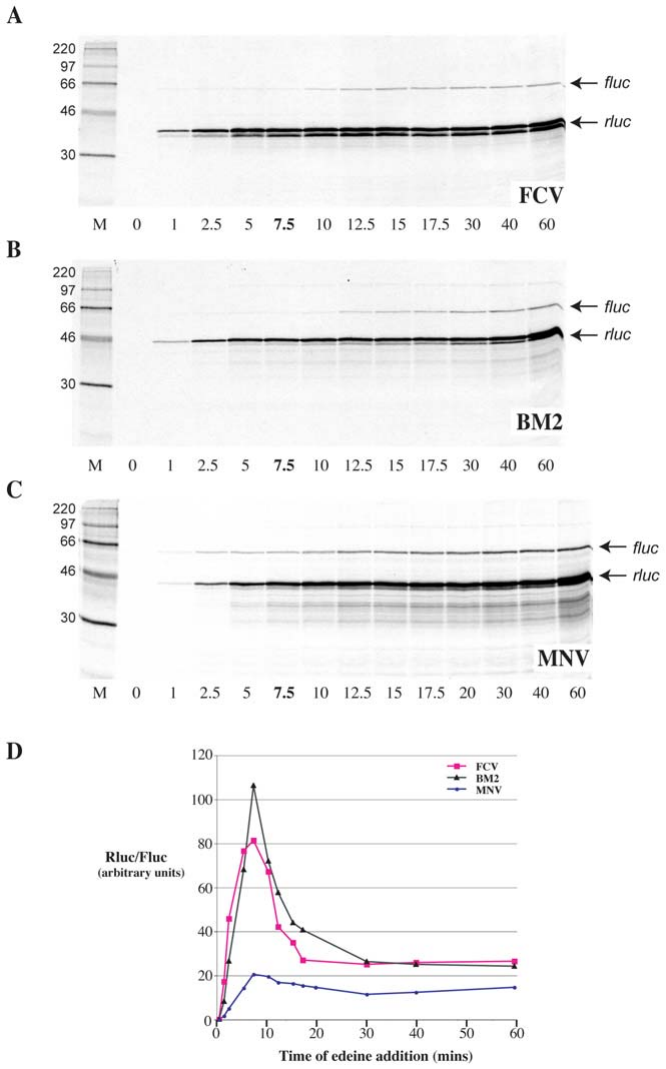
Effect of edeine on termination-reinitiation. Reporter mRNAs containing the termination reinitiation signals of FCV (panel A), BM2 (panel B) and MNV (Panel C) were translated in Flexi® RRL at a final RNA concentration of 50 µg/ml in the presence of [^35^S]-methionine and 140 mM added KCl. At the indicated time points (min), an aliquot was removed, edeine added to 5 µM, and the sample reincubated for a total of 60 min. The translation products were resolved by SDS-PAGE on 12% gels and visualised by autoradiography. Identical experiments were performed in which cycloheximide replaced edeine (data not shown). In the cycloheximide experiments, it was found that in all cases, no termination-reinitiation product was evident until the 7.5 min time point, when only a trace was visible. The 7.5 min time point in the edeine gels is emboldened to reflect this. The relative levels of the rluc and fluc bands was determined by densitometry and in Panel D, the Rluc/Fluc ratio is plotted against the time of edeine addition for the three mRNAs.

The precise mechanism of termination-reinitiation is not known, but the sensitivity of FCV VP2 protein expression to the translation initiation inhibitor edeine suggests that the reinitiation process bears at least some similarity to standard initiation at AUG codons [13]. To ascertain whether edeine sensitivity is a general feature of termination-reinitiation, we analysed the effect of the peptide on the activity of the MNV and BM2 signals (with FCV as a control) using translation time-courses (Figure 7). Reactions were programmed with the relevant mRNA and at various times an aliquot was removed, edeine added (to 5 µM) and the aliquot re-incubated such that the total time of translation was 60 minutes. To determine the time of first appearance of the termination and reinitiation products, identical reactions were also performed in which the elongation inhibitor cycloheximide replaced edeine. In the edeine experiments, it was evident that for FCV and BM2, only a trace of “stop-start” product was synthesised at the early time points. In these experiments, the vast majority of ribosomes did not reach the stop-start window until at least 7.5 minutes had passed (as shown in the cycloheximide time course experiments [data not shown; see legend to Figure 7]), thus the trace of VP2fluc seen likely corresponds to the product of infrequent internal initiation at the VP2fluc AUG or is derived from those few ribosomes that had reached the stop-start window prior to edeine addition. At later time points, however, the termination-reinitiation product steadily accumulated, with the ratio of the upstream and downstream ORFs stabilising after 30 minutes (at a reinitiation frequency of ∼4%). Thus for the FCV and BM2 signals, when edeine is present prior to arrival of ribosomes at the stop-start signal, it greatly inhibits termination-reinitiation, but has little effect on translation post-reinitiation. Unexpectedly, the MNV signal responded differently, with the termination-reinitiation product being more evident at early times post-edeine addition (in comparison to FCV and BM2). At these early time points, few ribosomes would have reached the stop-start window prior to edeine addition, thus the MNV signal shows increased resistance to the effects of edeine. Examination of the kinetics of synthesis of the two ORFs (Figure 7d) reveals that in all cases, the frequency of termination-reinitiation at early time points was higher than that seen at the steady state. This is indicative of a titration effect; early in the time course, when fewer ribosomes have loaded onto the mRNA (due to the earlier addition of edeine), the greater frequency of reinitiation may reflect the increased relative abundance of a necessary factor. The molecular basis of the resistance to edeine seen with the MNV signal is difficult to explain. It may be that recognition of the stop-start motif is indeed blocked by edeine but somehow, a proportion of initiation complexes still recognise the AUG present in the second pentanucleotide motif (UGAUG; see above) on the mRNA.

## Discussion

In this paper we show that expression *in vitro* of the murine norovirus VP2 protein occurs by coupled translation termination-reinitiation. The process requires the close proximity of stop and start codons, a defined region of mRNA upstream of the stop-start window that includes a functional TURBS Motif 1 and translation by the ribosome through this region up to the site of termination-reinitiation. Secondary structure mapping indicates that the RNA in this region is weakly structured, with Motif 1 loosely embedded in the 5′ arm of a putative stem-loop structure. The MNV signal thus exhibits many of the features and functional characteristics of the stop-start signals of FCV, RHDV and influenza B. The molecular mechanism of termination-reinitiation remains to be fully elucidated, however. Central to the discussion is the TURBS and in this context the purpose of the identified Motifs, the role (if any) of RNA secondary structure, and the functional requirement for translation through the TURBS.

**Figure 8:**
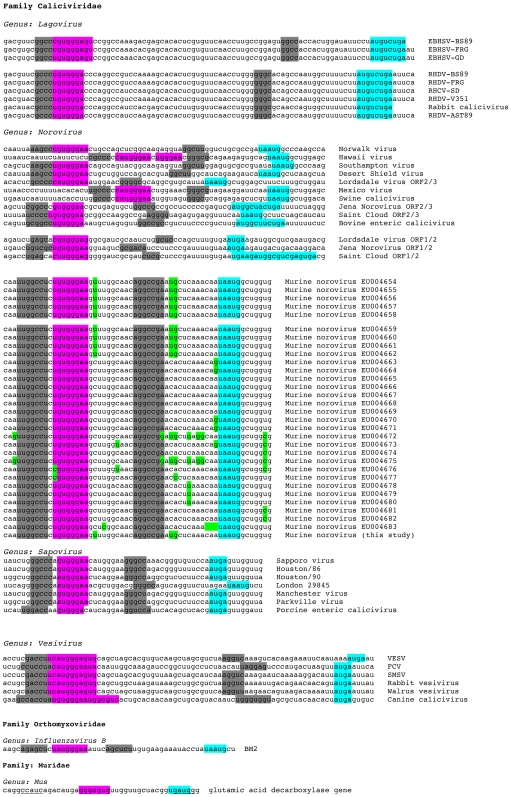
Comparison of caliciviral termination-reinitiation signals and 5′ flanking regions. The termination-reinitiation signal of influenza BM2 is also shown, as is a putative signal in the cellular gene glutamic acid decarboxylase [33]. Confirmed and potential Motif 1 sequences are highlighted in pink and the stop-start window in blue. Potential base-pairing interactions flanking Motif 1 [18] are indicated in grey (or underlined in the case of the glutamic acid decarboxylase gene). Within the murine noroviruses, in reference to EU004666, base changes are highlighted in green. Abbreviations used: EBHSV, European brown hare syndrome virus; RHDV, rabbit hemorrhagic disease virus; VESV, vesicular exanthema of swine virus; FCV, feline calicivirus; SMSV, San Miguel sealion virus.

Regarding Motif 1, it is clear that in all studies so far, mRNA mutations that would destabilise an interaction with 18S rRNA reduce or abolish reinitiation and changes not predicted to affect pairing having a lesser effect or none at all. Recently, the reciprocal experiment was performed, where mutations were introduced into the relevant region of (yeast) 18S rRNA. Their effect on termination-reinitiation was found to be highly consistent with a role for mRNA-18S rRNA pairing [18]. These experiments confirm a role in tethering through rRNA, although do not rule out the contribution of other factors, for example, binding of eIF3 [13]. A comparative alignment of the MNV signal with other known or suspected termination-reinitiation signals (Figure 8) reveals that Motif 1 is always present and that the stop and start codons of the termination-reinitiation site are in close proximity to each other. What does vary is the spacing between the two elements, from only 26 nt in the case of BM2 to 29 nt in MNV, 53 nt in FCV, 61 nt in RHDV and 62 nt (the longest) in the *Lagovirus* European brown hare syndrome virus. It is not clear whether the “additional” sequences present in viruses with longer TURBS have a role in termination-reinitiation. Deletion analysis of the FCV and RHDV TURBS has revealed some dispensible sequences - there may be some flexibility in the spacing of Motif 1 that allows other biological information to be accommodated into the TURBS without affecting function in stop-start. However, there is little sequence conservation between the signals of viruses of different genera, arguing against the presence of other primary sequence motifs. Another stretch of bases of functional consequence has been identified in FCV and RHDV, namely TURBS Motif 2, which is located closer to the stop-start window than Motif 1 and is speculated to help position ribosomes correctly at the reinitiation codon [12], [14]. Recent work has shown that the functional requirement for Motif 2 is in its participation in a base-paired region that forms between this motif and a stretch of bases immediately upstream of Motif 1 [18]; see Figure 8. This base-pairing has previously been noted from structure predictions of the signal of FCV [13] and direct RNA secondary structure probing of BM2 stem 2 [19] and the MNV stem 2 (see Figure 4). Based on the observations of Luttermann and Meyers [18], the formation of this stem is likely to be important to termination-reinitiation in the BM2 and MNV systems. Indeed, it is noticeable that in the deletion analysis of the MNV signal, and that of BM2 [19], those deletions that would affect formation of stem 2 showed reduced activity in termination-reinitiation (Figure 1b, Figure 4).

Despite this progress, the occurence and role of RNA secondary structure within viral TURBS is poorly understood. Direct structure probing and *mfold* analysis indicates that the RNA upstream of the stop-start window is metastable and whilst the secondary structures proposed for FCV [13], BM2 [19] and MNV (this study) are superficially similar, the largely single-stranded nature of the TURBS weakens these models and their comparison. The insertion of a premature termination codon upstream of the TURBS blocks reinitiation, ruling out the possibility that VP2 expression occurs by ribosome recruitment to a conventional, structured, viral IRES or by shunting from the untranslated region of the upstream ORF. The requirement for translation through the TURBS may simply reflect the need to deliver ribosomes to the stop-start window, but it could also indicate a requirement to remodel the TURBS, conceivably by alteration of RNA secondary structure or displacement of a bound factor. Based on chemical and enzymatic RNA structure probing of the BM2 signal and folding predictions (*mfold*), it has been suggested that transit of the ribosome to the stop-start window leads to melting of one stem-loop structure and the formation of an alternative structure that has Motif 1 displayed on its apical loop [19]. The position of MNV Motif 1 relative to the stop-start window is very similar to that of BM2 suggesting that the same remodelling could operate (Figures 4 and 8). However, whilst transit and termination of the ribosome would destabilise the identified secondary structure (Figure 4), liberating TURBS motif 1 in close proximity to helix 26, it is not clear whether this motif would subsequently be displayed as part of an alternative secondary structure. Whilst *mfold* analysis of the MNV region present locally upstream of the terminating ribosome does suggest an alternative secondary structure, further work will be needed to confirm this possibility.

Studies on the FCV signal have revealed that the reinitiation process occurs in the standard fashion by the criterion of sensitivity to edeine, but it is distinct in being completely independent of eIF4G or the eIF4F complex [13]. Analysis of the MNV signal here provides further evidence that the process deviates from the standard mechanism. First, like BM2 [19], there appears to be efficient use of non-AUG codons to reinitiate translation, indicating a relaxed requirement for the full complement of initiation factors, which would include eIF1 and eIF1A, thought to play important roles in locating and correct recognition of the AUG start codon [22]–[23]. Secondly, in contrast to what has been observed with FCV and BM2, the MNV signal is more resistant to treatment with edeine. Edeine does not inhibit binding of the eIF2/GTP/Met-tRNAi ternary complex to the 40S ribosomal subunit, nor Met-tRNAi/40S complex scanning, but there is a complete failure of AUG codon recognition, so that scanning continues past all AUG codons, and, probably as a secondary consequence, there is no ribosomal subunit joining [24]–[25]. The relative insensitivity of the MNV signal to edeine suggests that recognition of the AUG start codon during reinitiation may not require a scanning ternary complex. It is not clear why the FCV and BM2 signals respond differently to edeine, especially as the organisation of the BM2 signal (with regard to the position of Motif 1 and the primary sequence of the stop-start window) is so similar to that of MNV. Another observation that hints at non-standard reinitiation mechanisms relates to the the translation pattern seen with the MNV49.8 transcript. In this mRNA, the two termination-reinitiation windows (the natural UAAUG and the fortuitous downstream UGAUG) were mutated to eliminate the stop codon in each case. In translations of this mRNA, where termination occurs 30 amino acids downstream of the authentic site, very little termination-reinitiation product was seen, but an additional product was synthesised whose size is consistent with that of a fusion of the two reporter ORFs (asterisked in Figure 6). The origin of this protein is uncertain. It could have arisen through a ribosomal frameshift event, although no obvious conventional frameshift signals are present in the region of overlap between the two ORFs [26]–[27]. It could also represent the outcome of a failed attempt to terminate and subsequent resumption of translation by ribosomes on the downstream ORF. Further work will be required to elucidate the nature and origin of this product and how it relates to the mechanism of termination-reinitiation.

## Materials and Methods

### Construction of Plasmids

Plasmids used to assay termination-reinitiation were based on the p2luc reporter vector [20]. Sequences encompassing the stop-start signal of MNV (203 bp of sequence information upstream of the VP1 stop codon and 52 bp downstream) and FCV (97 bp upstream of the VP1 stop codon and 14 bp downstream) were generated by PCR (using Pfu polymerase [Roche]) from, respectively, plasmids pT7:MNV (kind gift of Dr Ian Goodfellow, Imperial College, London) and pSG-2/3* [13], a kind gift of Dr Tuiya Pöyry, University of Cambridge. The PCR products and p2luc were digested with *SalI* and *BamHI* and ligated together. Sequences were confirmed by dideoxy sequencing (using the facility at the Department of Biochemistry, University of Cambridge). The influenza B termination-reinitiation assay plasmid (p2luc-BM2wt; 250 bp upstream of M1 stop-codon, 18 bp downstream) was described previously [19].

### Site-Directed Mutagenesis

Site-directed mutagenesis was performed using the Quikchange II site-directed mutagenesis kit (Stratagene) according to manufacturer's instructions. For large deletions (greater than 48 bp) a modification of the manufacturer's protocol was used with the primers containing ∼30 bp of complementary sequence either side of the site of deletion, as described previously [28]. Mutagenesis to introduce insertions longer than 6 bp was performed in two steps [29], by first subjecting mutagenesis reactions (containing either the sense or antisense primer) to three cycles of PCR, then mixing the reactions and performing a further 18 cycles according to manufacturer's instructions.

### *In Vitro* Transcription and Translation

Reporter plasmids were linearised with *HpaI* and capped run-off transcripts generated using T7 RNA polymerase as described [30]. Messenger RNAs were recovered by a single extraction with phenol/chloroform (1∶1 *v/v*) followed by ethanol precipitation. Remaining unincorporated nucleotides were removed by gel filtration through a NucAway spin column (Ambion). The eluate was concentrated by ethanol precipitation, the mRNA resuspended in water, checked for integrity by agarose gel electrophoresis and quantified by spectrophotometry.

Unless otherwise stated, mRNAs were translated in Flexi® rabbit reticulocyte lysate (Flexi®RRL, Promega) programmed with 50 µg/ml template mRNA. Typical reactions were of 10 µl and composed of 60% (v/v) Flexi®RRL, 20 µM amino acids (lacking methionine), 500 µM MgOAc, 2 mM DTT, 5U RNAse inhibitor (RNAguard, GE Healthcare Life Sciences), 130 mM-160 mM KCl (optimised for each batch of Flexi®RRL) and 0.2 MBq [^35^S]-methionine. Reactions were incubated for 1 h at 30°C and stopped by the addition of an equal volume of 10 mM EDTA, 100 µg/ml RNase A followed by incubation at room temperature for 20 minutes. Samples were prepared for SDS-PAGE by the addition of 10 volumes of 2X Laemmli's sample buffer [31], boiled for 3 minutes and resolved on 12% SDS-PAGE gels. The relative abundance of products on the gels was determined by direct measurement of [^35^S]methionine incorporation using a Packard Instant Imager 2024.

### RNA Structure Mapping

A plasmid encoding the putative termination-reinitiation signal of MNV (p2luc-MNVwt) was modified by site-directed mutagenesis to include a T3 RNA polymerase promoter 30 bp upstream of the minimal required viral sequence generating plasmid p2luc-MNV-T3. RNA for structure mapping was prepared by *in vitro* transcription of *BamHI*-digested p2luc-MNV-T3 using T3 RNA polymerase. Transcription reactions were performed on a 200 µl scale essentially as described [30]. Structure mapping was performed using a 5′ end-labelling procedure as described previously [30], [32]. All probing reactions were performed in a final volume of 50 µl and contained ∼40,000 c.p.m. 5′ ^33^P-end-labelled transcript, 10 µg *Escherichia coli* rRNA and the relevant enzymatic or chemical probe. Further details are provided in the legend to Figure 3.
